# Two-Dimensional Transthoracic Echocardiography-Based Diagnosis of Right Ventricular Aneurysm: A Neglected Issue in Patients with Coronary Artery Disease: Case Series and Literature Review

**DOI:** 10.3390/diagnostics13132194

**Published:** 2023-06-28

**Authors:** Mohammadbagher Sharifkazemi, Zahra Rahnamun, Zehra Jumana, Shahdad Khosropanah

**Affiliations:** 1 Nemazee Hospital, Shiraz University of Medical Sciences, Shiraz 71364, Iran; 2 Cardiology Department, Tehran Heart Center, Tehran University of Medical Sciences, Tehran 31911, Iran

**Keywords:** heart ventricles, two-dimensional Echocardiography, right ventricular aneurysm

## Abstract

Right ventricular (RV) aneurysm is a very rare ventricular lesion. An aneurysm is formed mainly as a complication of myocardial infarction (MI). As an RV aneurysm is a potentially life-threatening occurrence, its appropriate diagnosis is of great significance. However, right-sided heart diseases, especially RV aneurysms, have been neglected for years. Recent studies in the literature have elucidated the role of the right side of the heart in patients’ prognosis and response to treatment. However, RV aneurysm has been scarcely investigated, and most of the attention has been given to the left ventricular aneurysm in patients with ischemic heart diseases (IHD). Herein, we investigated a total of 625 patients with IHD referred for two-dimensional transthoracic echocardiography (2D TTE), among whom 18 were diagnosed with RV aneurysms through precise examination of several TTE views. The characteristics of these cases, including demographics, medical history, and results of cardiac tests (which the patients underwent previously), were recorded and presented. This study emphasized the importance of performing a meticulous 2D TTE evaluation and a thorough examination of different views by an expert echocardiographer, with special attention to the presence of an RV aneurysm in a patient suffering from IHD who presented either with acute coronary syndrome, including MI, or chronic IHD. The scarcity of information, especially in terms of complications and the most appropriate diagnostic methods, calls for further studies in this regard.

## 1. Introduction

Cardiovascular diseases (CVDs), which include ischemic heart disease (IHD), heart failure (HF), stroke, and peripheral arterial disease, are the leading causes of global mortality, responsible for 17.8 million deaths and 35.6 million disabilities worldwide in 2017 [1]. About 80% of CVD-related mortalities occur in low- and middle-income countries [2]. Myocardial infarction (MI), defined as the ischemic necrosis of myocardial tissue, is a complex phenomenon that causes not only acute-phase death but also late-phase death, as patients who experience MI have a higher mortality rate in the first year after MI and in the years thereafter [3]. Post-MI complications include arrhythmias, heart block, cardiogenic shock, congestive HF, pericarditis, and mechanical complications such as ventricular aneurysm [4].

Ventricular aneurysms are diastolic as well as systolic outpouchings of the ventricle containing endocardium, epicardium, and thin-wall non-contractile scarred myocardium, which mainly develop in the setting of a full-thickness infarct, replaced by fibrous tissue because of MI. They occur most frequently in the left ventricle (LV) apical, anterior, and anteroseptal walls, and rarely in inferior-posterior or lateral walls [5]. Other causes of ventricular aneurysms include congenital, traumatic, infective, and idiopathic causes. Ventricular aneurysms occur predominantly in the LV, because of the greater myocardial volume and blood supply, and are associated with higher morbidity, complication rates, and greater in-hospital resource utilization [6]. A true ventricular aneurysm (involving the myocardium) can result in heart failure because of a reduction in the forward stroke volume, lethal ventricular arrhythmias, thromboembolism, and a low rate of rupture. Therefore, appropriate diagnosis and management and required [7].

**Figure 1:**
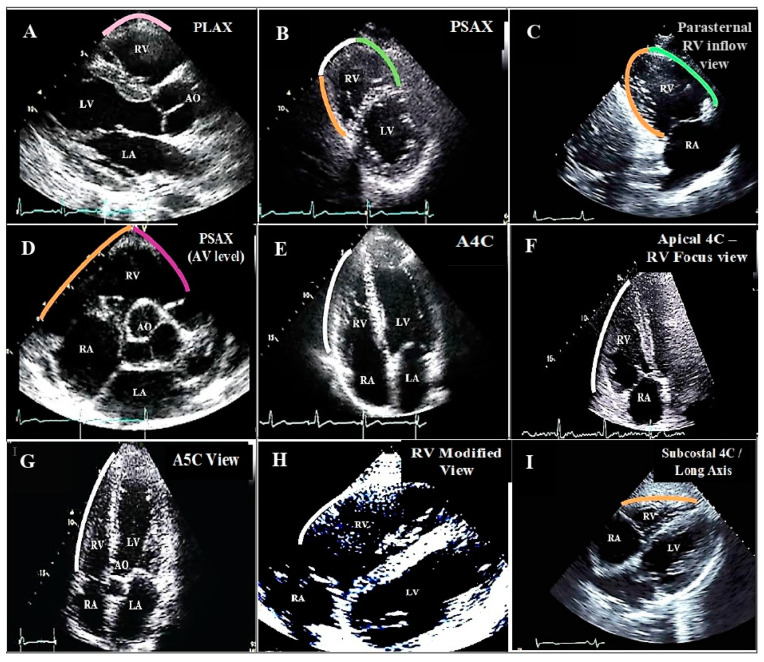
Different transthoracic and subcostal echocardiographic views displaying different right ventricular regions. 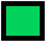 Anterior wall of the RV: supplied by acute marginal branches. 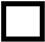 Lateral wall of the RV: supplied by acute marginal branches. 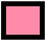 Anterior wall of the RVOT: supplied by conus branch. 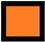 Inferior wall of the RV: supplied by the posterior descending artery. Abbreviations: A5C: apical five chamber; A4C: apical four chamber; AO: aorta; AV: aortic valve; LA: left atrium; LV: left ventricle; PLAX: parasternal long axis view (illustrating RVOT); PSAX: parasternal short axis view; RA: right atrium; RV: right ventricle; Subcostal 4C view: subcostal four chamber view. (**A**). Parasternal long axis view. (**B**). Parasternal Short axis view. (**C**). Parasternal RV inflow view. (**D**). Parasternal short axis view of great vessels. (**E**). Apical four chamber view. (**F**). Apical four chamber- RV focus view. (**G**). Apical five chamber view. (**H**). Parasternal right ventricular modified view. (**I**). Subcostal four chamber/long axis view.

Other causes of right ventricle (RV) aneurysms, such as congenital, traumatic, and right arrhythmogenic ventricular cardiomyopathy (ARVC), have also been reported [8,9,10] and some may remain undiagnosed until adulthood [11]. Development of ventricular aneurysms in the RV following infarction is a rare phenomenon [12,13,14,15], possibly due to the lower intraventricular pressure in the RV [16,17]. However, the different aspects of post-MI RV aneurysms have not been elucidated and more studies are required in this regard. The different transthoracic and subcostal echocardiographic views that display the blood supply in the different RV regions are shown in Figure 1.

Although electrocardiography (ECG) changes in favor of old infarction or ischemia usually remain for long periods and would be helpful when detecting the presence of LV aneurysms with high probable diagnostic accuracy [18], the chance of observing ECG changes as a diagnosis of RV aneurysm is zero. Accordingly, searching for signs of RV aneurysm during the conventional two-dimensional transthoracic echocardiography (2D TTE) can result in accurate diagnosis and appropriate treatment of RV aneurysms [19,20]; however, some have suggested that TTE is a less sensitive method and recommended more accurate cardiac imaging modalities such as cardiac magnetic resonance (CMR) and computed tomography (CT) scan [21]. More studies are required to determine the most appropriate diagnostic tool for RV aneurysms. Herein, we present the characteristics of 18 cases with RV aneurysms, successfully diagnosed by meticulous 2D TTE examination, among a total of 625 known cases of IHD/MI.

## 2. Case Presentation

From June 2020 to August 2021, a total of 625 patients with IHD were referred to the echocardiography department at our center for 2D TTE by a European accredited cardiologist echocardiographer. The echocardiographer examined several views, namely parasternal long axis, parasternal short axis (LV papillary muscle level and AV level), RV inflow, apical four and five chambers, RV focus and modified, and subcostal views. The diagnoses were confirmed by another expert cardiologist echocardiographer, who reviewed all recorded movies. The performance of 2D TTE, with special attention to RV, identified a total of 18 cases of RV aneurysms during this period.

The characteristics of the cases, including demographics, medical history, and results of cardiac tests that the patients had undergone previously were recorded and are presented in Table 1. As shown in this table, most of the 18 patients with RV aneurysms were men, with only six being women. The average age of patients was 65.94 years; two patients were young (32 and 35 years), and the oldest patients were 81 and 82 years old. Three patients presented with acute MI to the emergency department; however, old inferior MI was the most frequent finding in the ECG reports of patients.

The patients had a wide range of underlying diseases, including coronary artery disease (CAD, 15 patients), chronic kidney disease (10 patients, at different stages), hyperlipidemia (8 patients), hypertension (10 patients), and type II diabetes mellitus (T2DM, 5 patients); however, only 2 patients reported no underlying diseases. Most patients (*n* = 12) underwent coronary artery bypass graft (CABG) in the past, and another was indicated but refused to undergo CABG; 11 patients underwent percutaneous coronary intervention (PCI); three patients underwent the PCI procedure twice.

**Figure 2:**
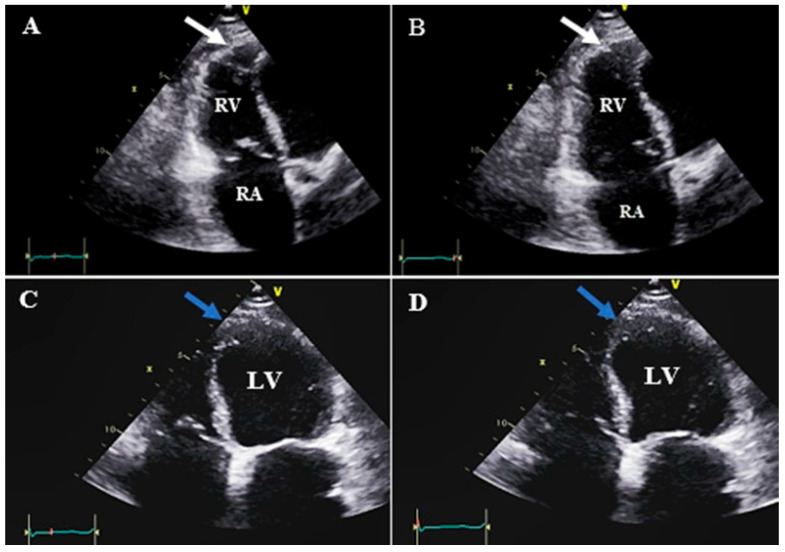
Right and left ventricular apical and apicolateral aneurysms in the apical RV focus view (white arrow) and apical four chamber view (blue arrow) with no clot (related to case #1); (**A**,**C**) end systole, (**B**,**D**) end diastole. Abbreviations: LV: left ventricle; RA: right atrium; RV: right ventricle.

**Figure 3:**
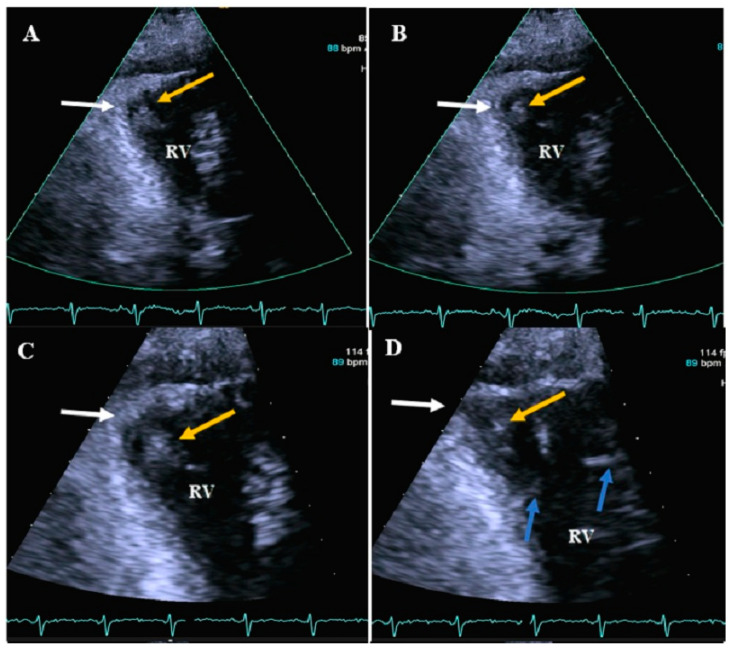

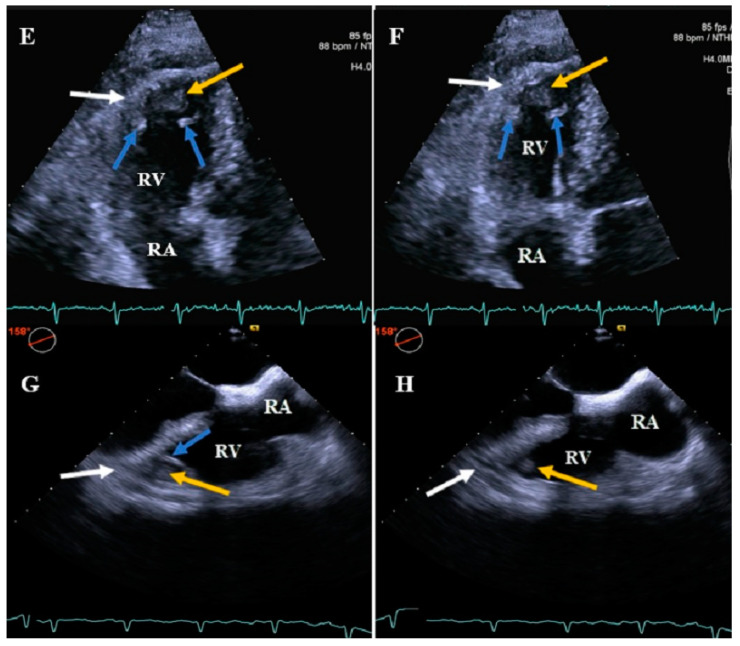
Right ventricular apical focus views (zoom-out and zoom-in images) of case #3 in end systole (**A**,**C**,**E**) and end diastole (**B**,**D**,**F**), illustrating apicolateral aneurysm (white arrow) accompanied by a semi-mobile thrombus (yellow arrow). The blue arrow shows the prominent moderator band. (**G**,**H**) show the two-dimensional midesophageal views in 158 degrees (**G**) in end systole and (**H**) in end diastole. The blue arrow shows a prominent moderator band, the white arrow shows the apicolateral aneurysm, and the yellow arrow shows a clot. Abbreviations: RA: right atrium; RV: right ventricle.

**Figure 4:**
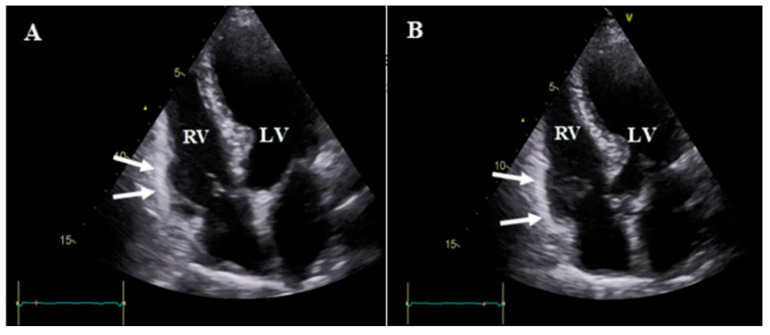
Apical right ventricular focus view of case #12 in end systole (**A**) and end diastole (**B**), illustrating RV aneurysm (white arrow) in the basal portion of the lateral wall. Abbreviations: LV: left ventricle; RV: right ventricle.

**Figure 5:**
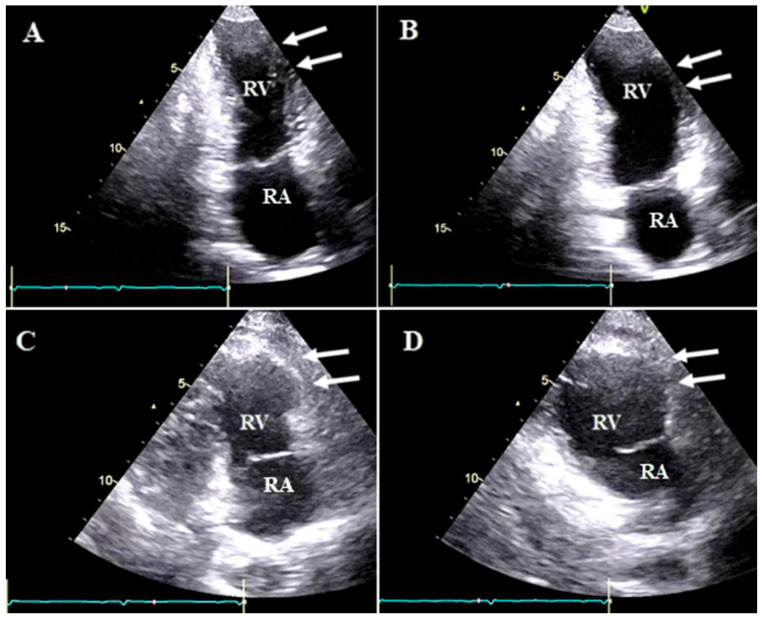
Apical right ventricular inflow views of case #13 in end systole (**A**,**C**) and end diastole (**B**,**D**), illustrating RV aneurysm (white arrow) in the mid-anterior portion. Abbreviations: RA: right atrium; RV: right ventricle.

**Figure 6:**
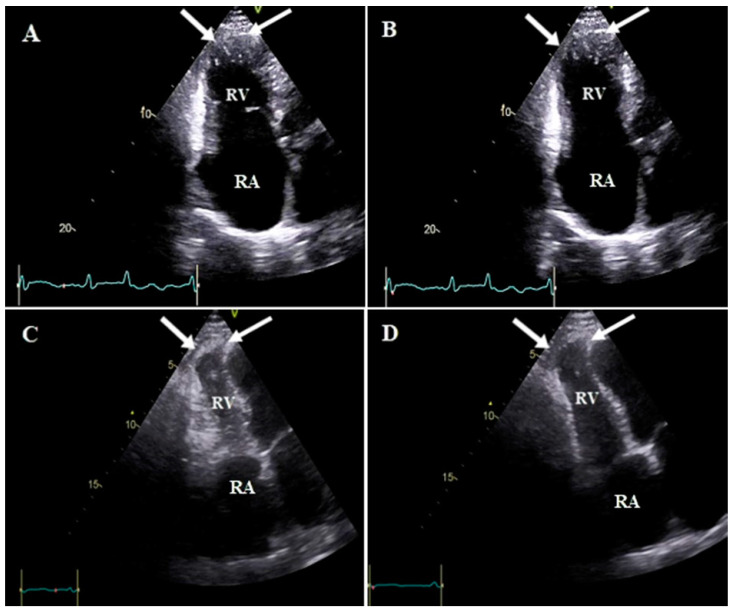
Modified apical four chamber and right ventricular apical focus views of case #16 in end systole (**A**,**C**) and end diastole (**B**,**D**), illustrating apical and apicolateral aneurysms (white arrow). Abbreviations: RA: right atrium; RV: right ventricle.

**Figure 7:**
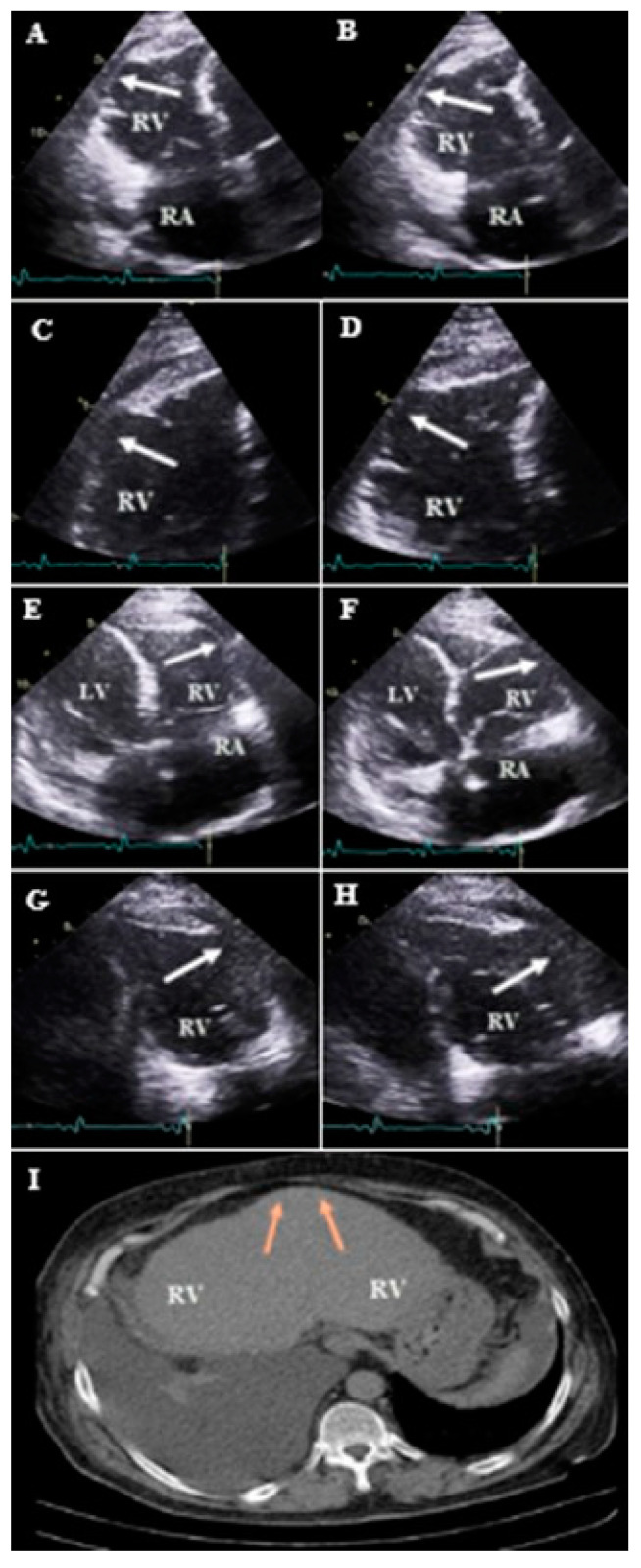
Apical RV focus views (zoom out and zoom in) illustrating thin and aneurysm in the mid free wall (white arrow) of case #18. (**A**,**C**) in end systole and (**B**,**D**) in end diastole. Reverse apical four chamber views (zoom out and zoom in) showing the same thin and aneurysm in mid RV free wall (white arrow). (**E**,**G**) in end systole and (**F**,**H**) in end diastole. High-resolution computed tomography of chest (**I**) illustrating thin and aneurysm in mid RV free wall (brown arrow). Abbreviations: LV: left ventricle; RA: right atrium; RV: right ventricle.

All patients had undergone coronary angiography, which indicated three-vessel disease in most patients, although two patients had two-vessel disease (case #15 and 16) and one patient had single-vessel disease (case #12). Right coronary artery (RCA) cut proximal to first acute marginal branch was observed in all except three patients (cases #5, 16, 17). LV systolic dysfunction was observed in all patients: nine severe dysfunctions (LVEF < 30%), five moderate (LVEF: 30–39%), and four mild (LVEF: 40–49%). Ten patients had LV aneurysms, four with clots. Thirteen patients had severe RV systolic dysfunction, while four had moderate RV systolic dysfunction. Three patients had RV aneurysms with a clot, while the rest had no clots. As the patients were referred to our center for 2D TTE, we could not follow them up and had no information on the patients’ conditions after TTE. The important imaging findings, including 2D TTE in cases 1, 3, 12, 13, 16, and 17 are presented in Figure 2, Figure 3, Figure 4, Figure 5, Figure 6 and Figure 7, and the rest of the cases’ imaging findings are presented as supplementary figures (Figures S1–S12). The supplementary video also shows the results of all 18 patients’ echocardiography, demonstrating RV aneurysms.

## 3. Discussion

Identification of 18 cases of RV aneurysms using 2D TTE among 625 patients with a positive history of IHD in the present study suggests that RV aneurysms are not very uncommon in this group of patients, although the present study was not an epidemiological study and cannot be used to make a conclusion on the frequency or rate of the disease. A series published in 1991 reported diagnoses of 12 cases of RV aneurysm among 137 patients with acute MI (8.75%) who were diagnosed using radionuclide angiocardiography [22]. This frequency is even higher than that reported in the present series, possibly due to the fact that they did not consider patients with acute MI, while we considered patients with a diagnosis of chronic IHD, among whom only three had a recent acute MI. However, we may not be able to compare them with the present series, as CVD-related risk factors, diagnostic methods, and therapeutic interventions have changed significantly since then. One case report was also reported [12,13,14,15]. Therefore, further reviews or epidemiological studies are required to estimate the incidence of RV aneurysm and its different aspects in the presence of acute MI and chronic IHD. The first case of isolated post-infarct RV aneurysm was reported in 1987 as a postmortem finding [15]. Besides the case series mentioned above (1991) [22], another case was reported in 2001 in a patient with acute inferior-posterior MI [14]. A third case reported in 2004 was of a 66-year-old man who was referred with post-MI angina (inferior MI 21 and 15 years and anteroseptal MI 2 years before) who underwent CABG for the three-vessel disease and was diagnosed with RV aneurysm using TTE and CMR 15 days after surgery [12]. The case presented by Ortoleva and colleagues was a 71-year-old man diagnosed with RV MI, whose RV aneurysm was diagnosed during surgery; the patient died 11 days after infarct [13]. The two later cases of post-MI RV aneurysms may not be a definite result of MI, as the diagnosis of RV aneurysm was reported after or during CABG, which might suggest potential aneurysm development because of surgical injury rather than purely the result of MI-related injuries [12,13,17]. Although most of our patients (*n* = 12) had undergone CABG, the procedure was performed years prior (CABG was performed a week prior in only one case) and traumatic origin is not presumed in these patients.

Other etiologies have also been associated with RV aneurysms, and there are frequent reports of RV aneurysms in different settings, including cardiomyopathy [23], traumatic [24], constrictive pericarditis [25], and sarcoidosis [26]. Furthermore, some cases have reported RV pseudoaneurysm (with no myocardium layer) [27,28], which is a different presentation and was therefore not discussed here. The risk factors for post-MI RV aneurysms are also not yet clear. In the present study, we observed concomitant development of RV aneurysms and CKD, hyperlipidemia, and hypertension. The association between CAD and CVD-related risk factors (smoking, hyperlipidemia, hypertension, and T2DM) in patients with a history of MI is predictable. Impaired renal function has also been previously suggested as a risk factor for LV aneurysms [29]. As CKD is a significant predictor of worse prognosis in patients with CAD and cardiac interventions can impair renal function, there seems to be a relationship between CAD and CKD [30,31]. As CKD (different stages) was observed in 10 out of 18 patients in our series, more studies are required to investigate the association between CKD and RV aneurysms.

Generally speaking, when MI is discussed in the clinical setting, LV is the focus of attention during clinical and paraclinical cardiac examinations, including ECG and TTE, which results in a lower diagnosis of RV pathologies. Nevertheless, as recent evidence has emphasized, RV MI is present in more than half of patients with LV MI, resulting in poorer patient prognosis and more major cardiac events [32]. Presumably, similar to LV aneurysms, RV aneurysms may also result in thrombosis, rupture, and sudden death [33,34,35]. Moreover, many cases of RV aneurysms are associated with LV aneurysms, which worsen patients’ prognosis; therefore, in patients experiencing MI, it is necessary to examine the signs of inferior MI and RV MI on ECG reports and echocardiographic examinations [36]. Similarly, when a ventricular aneurysm is discussed, LV is often the focus of attention, and most cases in the literature have focused on LV aneurysms [6], yet RV aneurysm is also of great significance and should be considered. However, RV is generally considered the forgotten chamber. Recent research has focused on RV and the significance of RV MI. As RV MI can occur with or without LV MI, it is expected that post-MI RV aneurysms also occur with or without LV aneurysms. Our experience emphasized that searching for RV aneurysms in different views and performing a thorough examination of different 2D TTE views by an expert cardiologist echocardiographer revealed the presence of LV/RV aneurysms in half of the patients (9/18) with chronic IHD/MI. Evidence of the presence of RV aneurysms has been found in patients with arrhythmogenic RV cardiomyopathy [37]; however, its presence in patients with acute or chronic IHD has rarely been discussed. Therefore, careful examination of both ventricles for the presence of aneurysms in suspected cases and training the fellow and technician echocardiographers on the diagnostic methods for RV aneurysm using 2D TTE are recommended. Similarly, some researchers have reported diagnosis of RV aneurysms using conventional TTE [19,20], while others have claimed that TTE is an insensitive tool and results in missed diagnosis and have therefore suggested more accurate cardiac imaging modalities [21]. Further studies are required to compare the accuracies of different diagnostic modalities and to recommend the most appropriate one.

Because of the scant evidence on MI-related RV aneurysms, the complications and adverse events associated with this condition are not clearly defined but may be similar to those associated with LV aneurysms, during which the thin-walled ventricle cannot contract appropriately during systole (against the pulmonary artery) and herniates outward (out pouch). The paradox contractility and dyskinesia caused by the aneurysm result in malignant arrhythmia, HF, and sudden cardiac death [38]. Thrombus formation is one of the important complications of ventricular aneurysm, most probably formed by blood stasis caused by the dyskinetic region of the ventricle, endothelial injury, and hypercoagulable state, i.e., the Virchow’s triad caused by MI [39]. As shown in the present study, four patients had thrombus in the LV and three had thrombus in the RV because of the aneurysm, which is of great significance as it can result in systemic thromboembolic events and probably pulmonary embolism in the absence of adequate management [39,40]. Therefore, it is necessary to diagnose ventricular aneurysms, especially the rare RV aneurysm, in order to apply the appropriate treatment and preventive strategy to improve prognosis. Additionally, more studies on treatment strategies and their efficacies on patients’ prognoses are required.

## 4. Conclusions

All in all, right ventricular aneurysm is potentially a very rare complication of MI, and the few case reports available in the literature have not determined the exact complications and prognosis, nor the most appropriate diagnostic modalities and treatment strategies. As ventricular aneurysms can be fatal, appropriate diagnosis and treatment are necessary. It is important to keep in mind that RV aneurysm is not a very rare condition and should not be neglected in the echo labs, as well as in the current literature. We believe that diagnosis of RV aneurysm is possible using the currently available and cost-effective cardiac imaging performed routinely in patients with a history of IHD. Therefore, cardiologists should pay greater attention to the hallmarks suggestive of RV aneurysm and train echocardiograph fellows and technicians on this regard as well. Furthermore, prospective research studies are required to accurately determine the incidence rate of RV aneurysm and the most accurate diagnostic method and to suggest the most appropriate treatment strategy.

## Figures and Tables

**Table 1:** Summary of patients’ characteristics, medical histories, and results of cardiac tests.

N.	Sex	Age	Underlying Diseases	Smoker	ECG Result	Vessel Disease	RCA Cut Proximal to RV Branch	Stage of LV Systolic Dysfunction	Lv Aneurysm	LV Clot	Stage of RV Systolic Dysfunction	Anatomical Location of RV Aneurysm in TTE	RV Clot	Other Findings
1	M	68	HLP, HTN, CAD, CKD stage II, CABG, PCI	+	OIMI	3	+	Moderate	+	-	Severe	apical and apicolateral	-	-
2	M	58	HLP, CAD, CKD stage III, CABG	+	OIMI	3	+	Severe	+	+	Severe	distal part of RVOT	-	DHF
3	F	82	HLP, HTN, CAD, T2DM, CKD stage II, PCI	-	OIMI	3	+	Severe	-	-	Severe	apicolateral	+	-
4	M	62	CAD, CABG	+	OIMI	3	+	Severe	-	-	Severe	mid lateral wall	-	-
5	F	74	HLP, HTN, T2DM, CAD, CKD stage III, CABG, PCI	-	OIMI	3	-	Moderate	+	-	Severe	apicolateral	+	DHF
6	F	81	HTN, CAD, CKD stage II, refused to undergo CABG	+	OIMI	3	+	Severe	+	+	Severe	basal portion of anterior wall	-	-
7	M	69	HLP, HTN, T2DM, CAD, CKD stage IV, CABG, PCI	+	OIMI	3	+	Severe	+	-	Severe	mid portion of lateral wall	-	DHF
8	F	66	HLP, HTN, CAD, CKD stage II, CABG, PCI	-	NS	3	+	Severe	+	-	Severe	basal portion of lateral wall	-	-
9	M	60	CAD, CABG last week	+	NS	3	+	Mild	-	-	Moderate	apicolateral	-	-
10	M	35	Family history of CAD	+	Acute inferoposterior+RV MI	3	+	Mild	-	-	Moderate	apical and apicolateral	+	Received fibrinolytic therapy
11	M	69	Chronic ischemic cardiomyopathy, CABG	+	OIMI	3	+	Severe	+ apical	+	Severe	distal part of RVOT	-	Candidate for ICD
12	M	32	-	+	Acute inferoposterior and RV MI	1	Severe proximal RCA thrombosis	Mild	-	-	Severe, akinesia+ mild RV dilation	basal portion of lateral wall	-	Acute chest pain
13	F	76	HTN, CAD, CKD stage II, CABG, PCI	+	Old inferior STEMI	3	+	Moderate, akinetic inferior walls	-	-	Moderate, mild RV dilation	mid-anterior portion	-	stable angina
14	M	74	HLP, HTN, CAD, CKD stage II, CABG, PCI	+	OIMI	3	+	Moderate	+ apical	-	Severe	basal portion of inferior wall	-	angina pectoris for months
15	M	79	HTN, T2DM, CAD, CKD stage IV, PCI twice	+	NS	2	+	Severe	+	+ apical	Severe	basal lateral wall	-	-
16	M	60	CAD, CABG, PCI twice	+	OIMI	2	+ cut mid RCA	Moderate	+ apical	-	moderate	apical and apicolateral	-	-
17	F	76	CAD, PCI	-	Acute inferior MI	3	+, conus branch stenosis plus cut mid RCA	Mild	-	-	Mild	distal part of RVOT plus mid anterior wall	-	Planned for staged CABG
18	M	65	CAD, PCI twice, HTN, T2DM, HLP	+	OIMI	3	+	Severe	-	-	Severe	mid RV free wall	-	DHF

Abbreviations: CABG: coronary artery bypass graft; CAD: coronary artery disease; CKD: chronic kidney disease; DHF: decompensated heart failure; HLP: hyperlipidemia; HTN: hypertension; ICD: implantable cardioverter defibrillator; LAD: left anterior descending artery; LCX: left circumflex; LV: left ventricle; MI: myocardial infarction; NS: non-significant; OIMI: old inferior myocardial infarction; DHF: diastolic heart failure; PCI: percutaneous coronary intervention; RCA: right coronary artery; RV: right ventricle; STEMI: ST elevation myocardial infarction; T2DM: type II diabetes mellitus.

## Data Availability

The raw data supporting the conclusions in this article will be made available by the authors without undue reservation.

## Supplementary Materials

The following supporting information can be downloaded at: https://www.mdpi.com/article/10.3390/diagnostics13132194/s1, Figure S1: Findings of 2D TTE and coronary angiography in case #2. Parasternal long axis and off-axis parasternal long-axis views in end-systole (A,C,E) and end-diastole (B,D,F), illustrating right ventricular apical akinesia to dyskinesia (yellow arrow) with right ventricular outflow tract aneurysm (white arrow). (G,H). Coronary angiography showing cut RCA from mid part plus originating conus branch from a diseased part of proximal RCA filled by a white plaque (G; yellow arrow) and advanced disease in the left coronary system (H). Abbreviations: LV; left ventricle, RVOT; right ventricular outflow tract; RCA; right coronary artery; Figure S2: Findings in different views of 2D TTE in case #4. Apical RV Focus View, showing RV apical and apicolateral akinesia to dyskinesia (white arrow) with no clot (A,B), RV modified view showing mid-lateral wall aneurysm (yellow arrow; C,D), Apical four chamber view (E,F), showing dilated four chambers with LV apical segments aneurysm and a large size clot protruding into the cavity (blue arrow), Apical 2 and 3 chambers views (G and H, respectively), which illustrate thin and akinetic bases of inferior and posterolateral walls plus a part of the same protruding clot in the cavity (blue arrow). A,C,E,G in the end systole; B,D,F,H in the end diastole. Abbreviations: LV; left ventricle, RV; right ventricle, RA; right atrium; Figure S3: Findings in different views of 2D TTE in case #5. Parasternal long and off-axis views (A,B), illustrating RV apical akinesia to dyskinesia (green arrow) and apicolateral aneurysm (white arrow) with a layered clot (yellow arrow), RV modified view (C,D), showing RV apical akinesia to dyskinesia (green arrow) and apicolateral aneurysm (white arrow) with a layered clot (yellow arrow), Modified apical four chamber view (E,F), showing LV apical segments aneurysm, in addition to an apical layered clot (blue arrow). Abbreviations: LV; left ventricle, RV; right ventricle, RVOT; right ventricular outflow tract; Figure S4: Modified apical four chamber view of case #6, illustrating RV aneurysm (white arrow) in the basal portion of anterior wall; A. in end-systole, B. in end-diastole. Abbreviations: LV; left ventricle, PE; pericardial effusion, RA; right atrium, RV; right ventricle; Figure S5: RV modified view of case #7, illustrating RV aneurysm (white arrow) in the mid portion of lateral wall; A. in end-systole, B. in end-diastole. Abbreviations: AO; aorta, RV; right ventricle; Figure S6: Findings of 2D TTE and coronary angiography in case #8. Apical four chamber view, illustrating RV aneurysm (white arrow) in the basal portion of lateral wall; A. in end-systole, B. in end-diastole. Right coronary angiography (yellow arrow) shows significant narrowing in the osteoproximal portion of the second AMB (black arrow); C. in the 45-degree LAO projection, D. in AP cranial projection. RV cineangiography in 45-degree LAO projection illustrating aneurysm in basal portion (red arrow); E, in diastole, F in systole. Abbreviations: AMB; acute marginal branch, LV; left ventricle, RV; right ventricle, LAO; left anterior oblique; Figure S7: Findings of 2D TTE and coronary angiography in case #9. Aneurysms of RV apicolateral and bases of LV inferior and posterior walls (white and yellow arrows, respectively); A, C, and E in the end-systole; B,D,F in the end- diastole. Right coronary angiography (light purple arrow) before (G) and after (H) RCA PPCI, NB: the acute marginal branch (black arrow) was compromised during PPCI. Abbreviations: LV; left ventricle, RV; right ventricle; Figure S8: Findings of different 2D TTE views in case #10. A–C. Parasternal long axis view, illustrating RV apical and apicolateral aneurysm (white arrow), accompanied with a layered 1.5 cm^2^ clot (yellow arrow), D,E. Apical modified four chamber views, showing RV apical and apicolateral aneurysms (white arrow) plus mid anterior wall (green arrow), F,G. Apical four chamber view, showing LV apical aneurysm (blue arrow) and RV apical aneurysm (white arrow), H and I. RV apical focus view, showing RV apical and apicolateral aneurysm (white arrow). A,C,D,F,H in the end systole B,E,G,I in the end diastole. Abbreviations: LV; left ventricle, RV; right ventricle, RVOT; right ventricular outflow tract; Figure S9: Modified parasternal long axis view of case #11, illustrating aneurysm in the distal part of RVOT (white arrow), with basal LV posterior aneurysm (blue arrow); A. in end-systole, B. in end-diastole. Abbreviations: LV; left ventricle, RVOT; right ventricular outflow tract; Figure S10: Subcostal four chamber view of case #14, illustrating RV aneurysm (yellow arrow) in the basal portion of inferior wall; A. in end-systole, B. in end-diastole. Abbreviations: LV; left ventricle, RV; right ventricle; Figure S11: RV basal lateral wall aneurysm in the apical RV focus view of case #15: (white arrow) with no clot, illustrating LV basal inferior wall aneurysm in the apical two chamber view (blue arrow); A and C. in end systole, B,D. in end diastole. Abbreviations: LV; left ventricle, RA; right atrium, RV; right ventricle; Figure S12: Findings of 2D TTE and coronary angiography in case #17. Parasternal long axis view (A,B), illustrating distal RVOT aneurysm (white arrow). Parasternal RV inflow view (C,D), showing mid anterior wall aneurysm (light brown arrow). Right coronary cineangiography (E,F), showing significant narrowing in the conus branch ostia, in addition to diseased and cut RCA from mid part (blue arrow) in 45-degree left anterior oblique projection. Abbreviations: LV; left ventricle, RA; right atrium, RV; right ventricle, RVOT; right ventricular outflow tract; Video S1: Results of all 18 patients’ echocardiography, demonstrating RV aneurysms.
