# Supplementary material for: Two-Dimensional Transthoracic Echocardiography-Based Diagnosis of Right Ventricular Aneurysm: A Neglected Issue in Patients with Coronary Artery Disease: Case Series and Literature Review
Source: Diagnostics (Basel). 2023 Jun 28;13(13):2194. doi: 10.3390/diagnostics13132194 (PMC10340304; doi:10.3390/diagnostics13132194)
Supplement: Supplementary file 1 [file diagnostics-13-02194-s001.zip › Supplementary figure legends.pdf]

Supplementary figure legends:

Figure S1. Findings of 2D TTE and coronary angiography in case #2. Parasternal long axis and off-axis parasternal long-axis views in end-systole (A, C, and E) and end-diastole (B, D, and F), illustrating right ventricular apical akinesia to dyskinesia (yellow arrow) with right ventricular outflow tract aneurysm (white arrow). G and H. Coronary angiography showing cut RCA from mid part plus originating conus branch from a diseased part of proximal RCA filled by a white plaque (G; yellow arrow) and advanced disease in the left coronary system (H). Abbreviations: LV; left ventricle, RVOT; right ventricular outflow tract; RCA; right coronary artery.

Figure S2. Findings in different views of 2D TTE in case #4. Apical RV Focus View, showing RV apical and apicolateral akinesia to dyskinesia (white arrow) with no clot (A and B), RV modified view showing mid-lateral wall aneurysm (yellow arrow; C and D), Apical four chamber view (E and F), showing dilated four chambers with LV apical segments aneurysm and a large size clot protruding into the cavity (blue arrow), Apical 2 and 3 chambers views (G and H, respectively), which illustrate thin and akinetic bases of inferior and posterolateral walls plus a part of the same protruding clot in the cavity (blue arrow). A, C, E, and G in the end systole; B, D, F, and H in the end diastole. Abbreviations: LV; left ventricle, RV; right ventricle, RA; right atrium.

Figure S3. Findings in different views of 2D TTE in case #5. Parasternal long and off-axis views (A and B), illustrating RV apical akinesia to dyskinesia (green arrow) and apicolateral aneurysm (white arrow) with a layered clot (yellow arrow), RV modified view (C and D), showing RV apical akinesia to dyskinesia (green arrow) and apicolateral aneurysm (white arrow) with a layered clot (yellow arrow), Modified apical four chamber view (E and F), showing LV apical segments

aneurysm, in addition to an apical layered clot (blue arrow). Abbreviations: LV; left ventricle, RV; right ventricle, RVOT; right ventricular outflow tract.

Figure S4. Modified apical four chamber view of case #6, illustrating RV aneurysm (white arrow) in the basal portion of anterior wall; A. in end-systole, B. in end-diastole. Abbreviations: LV; left ventricle, PE; pericardial effusion, RA; right atrium, RV; right ventricle.

Figure S5. RV modified view of case #7, illustrating RV aneurysm (white arrow) in the mid portion of lateral wall; A. in end-systole, B. in end-diastole. Abbreviations: AO; aorta, RV; right ventricle.

Figure S6. Findings of 2D TTE and coronary angiography in case #8. Apical four chamber view, illustrating RV aneurysm (white arrow) in the basal portion of lateral wall; A. in end-systole, B. in end-diastole. Right coronary angiography (yellow arrow) shows significant narrowing in the osteoproximal portion of the second AMB (black arrow); C. in the 45-degree LAO projection, D. in AP cranial projection. RV cineangiography in 45-degree LAO projection illustrating aneurysm in basal portion (red arrow); E, in diastole, F in systole. Abbreviations: AMB; acute marginal branch, LV; left ventricle, RV; right ventricle, LAO; left anterior oblique.

Figure S7. Findings of 2D TTE and coronary angiography in case #9. Aneurysms of RV apicolateral and bases of LV inferior and posterior walls (white and yellow arrows, respectively); A, C, and E in the end-systole; B, D, and F in the end- diastole. Right coronary angiography (light purple arrow) before (G) and after (H) RCA PPCI, NB: the acute marginal branch (black arrow) was compromised during PPCI. Abbreviations: LV; left ventricle, RV; right ventricle.

Figure S8. Findings of different 2D TTE views in case #10. A, B, & C. Parasternal long axis view, illustrating RV apical and apicolateral aneurysm (white arrow), accompanied with a layered 1.5

cm^2 clot (yellow arrow), D and E. Apical modified four chamber views, showing RV apical and apicolateral aneurysms (white arrow) plus mid anterior wall (green arrow), F and G. Apical four chamber view, showing LV apical aneurysm (blue arrow) and RV apical aneurysm (white arrow), H and I. RV apical focus view, showing RV apical and apicolateral aneurysm (white arrow). A, C, D, F, and H in the end systole B, E, G, and I in the end diastole. Abbreviations: LV; left ventricle, RV; right ventricle, RVOT; right ventricular outflow tract.

Figure S9. Modified parasternal long axis view of case #11, illustrating aneurysm in the distal part of RVOT (white arrow), with basal LV posterior aneurysm (blue arrow); A. in end-systole, B. in end-diastole. Abbreviations: LV; left ventricle, RVOT; right ventricular outflow tract.

Figure S10. Subcostal four chamber view of case #14, illustrating RV aneurysm (yellow arrow) in the basal portion of inferior wall; A. in end-systole, B. in end-diastole. Abbreviations: LV; left ventricle, RV; right ventricle.

Figure S11. RV basal lateral wall aneurysm in the apical RV focus view of case #15: (white arrow) with no clot, illustrating LV basal inferior wall aneurysm in the apical two chamber view (blue arrow); A and C. in end systole, B and D. in end diastole. Abbreviations: LV; left ventricle, RA; right atrium, RV; right ventricle,

Figure S12. Findings of 2D TTE and coronary angiography in case #17. Parasternal long axis view (A and B), illustrating distal RVOT aneurysm (white arrow). Parasternal RV inflow view (C and D), showing mid anterior wall aneurysm (light brown arrow). Right coronary cineangiography (E and F), showing significant narrowing in the conus branch ostia, in addition to diseased and cut RCA from mid part (blue arrow) in 45-degree left anterior oblique projection. Abbreviations: LV; left ventricle, RA; right atrium, RV; right ventricle, RVOT; right ventricular outflow tract.
