# Supplementary figures and images for: Two-Dimensional Transthoracic Echocardiography-Based Diagnosis of Right Ventricular Aneurysm: A Neglected Issue in Patients with Coronary Artery Disease: Case Series and Literature Review
Source: Diagnostics (Basel). 2023 Jun 28;13(13):2194. doi: 10.3390/diagnostics13132194 (PMC10340304; doi:10.3390/diagnostics13132194)

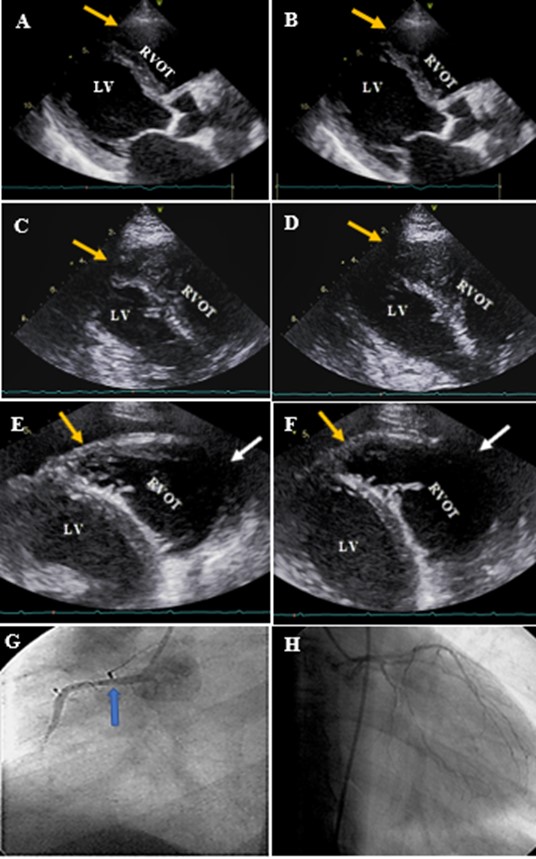

Supplement: Supplementary file 1 [file diagnostics-13-02194-s001.zip › figure S1-case 2.jpg]

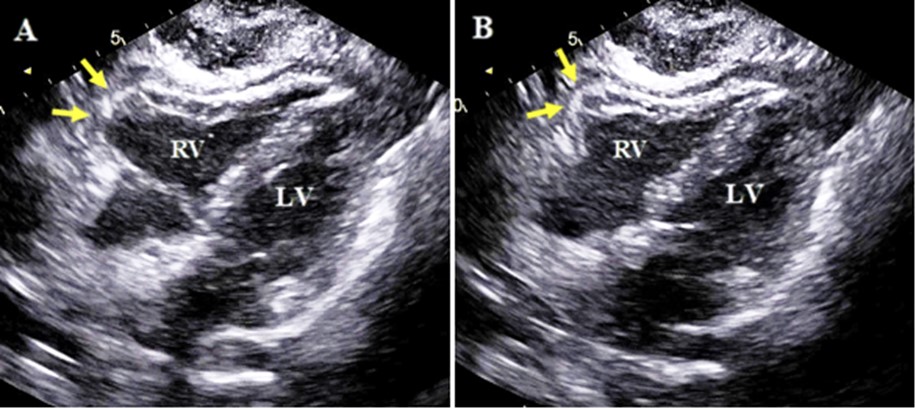

Supplement: Supplementary file 1 [file diagnostics-13-02194-s001.zip › figure S10-case 14.jpg]

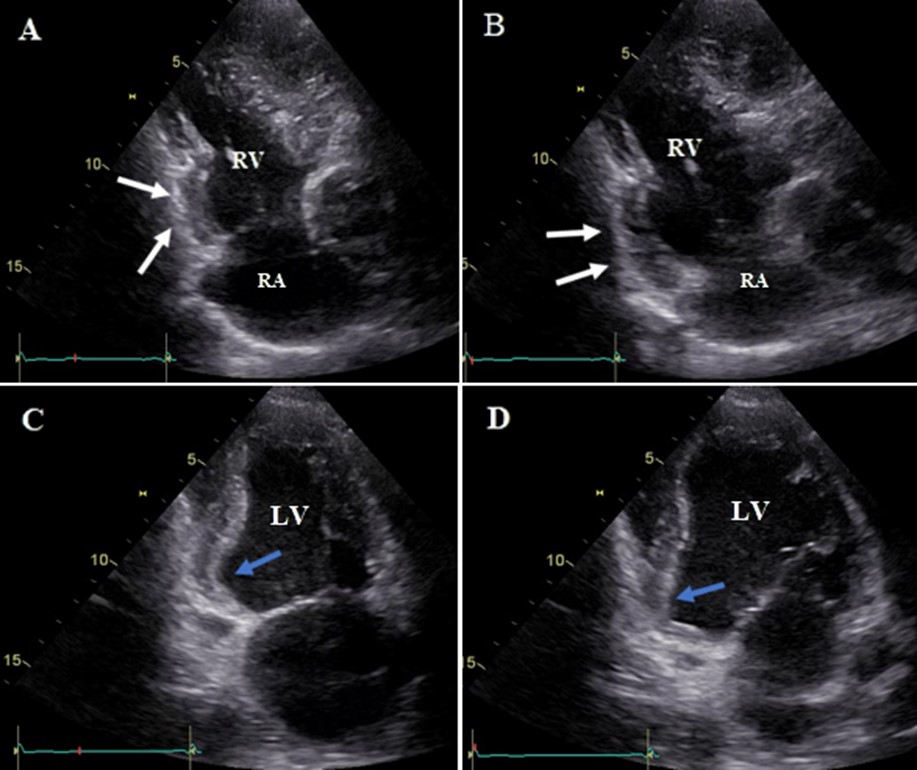

Supplement: Supplementary file 1 [file diagnostics-13-02194-s001.zip › figure S11-case 15.jpg]

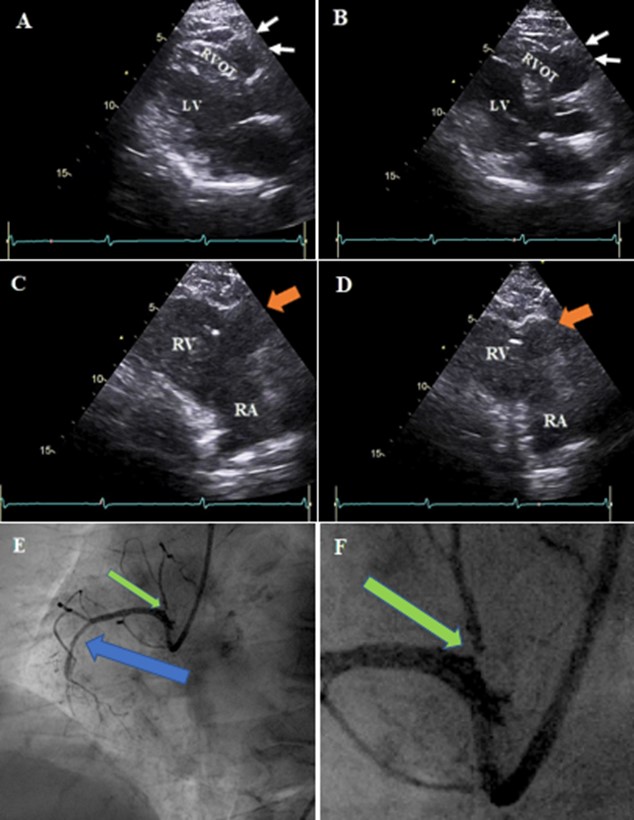

Supplement: Supplementary file 1 [file diagnostics-13-02194-s001.zip › figure S12-case 17.jpg]

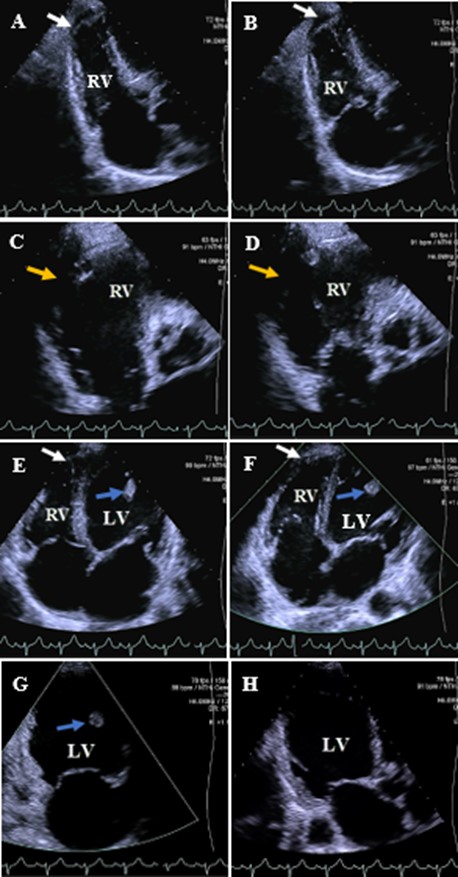

Supplement: Supplementary file 1 [file diagnostics-13-02194-s001.zip › figure S2-case 4.jpg]

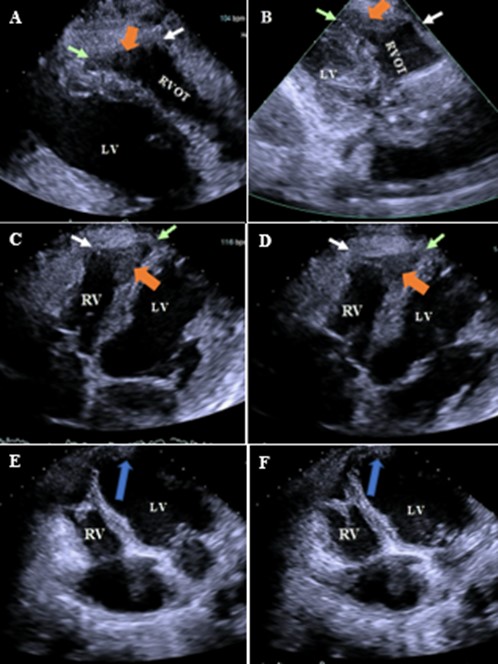

Supplement: Supplementary file 1 [file diagnostics-13-02194-s001.zip › figure S3-case 5.jpg]

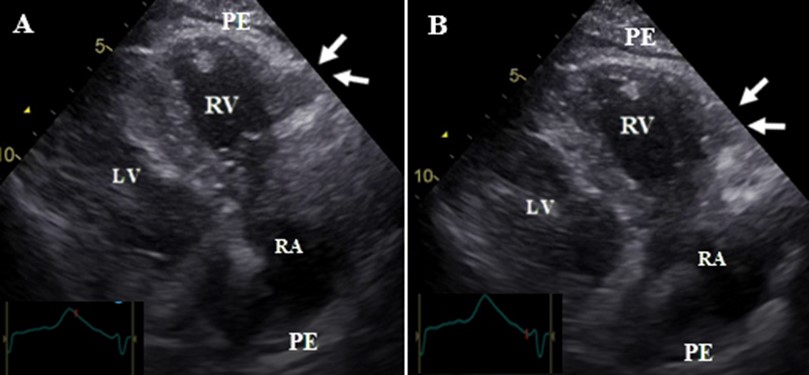

Supplement: Supplementary file 1 [file diagnostics-13-02194-s001.zip › figure S4-case 6.jpg]

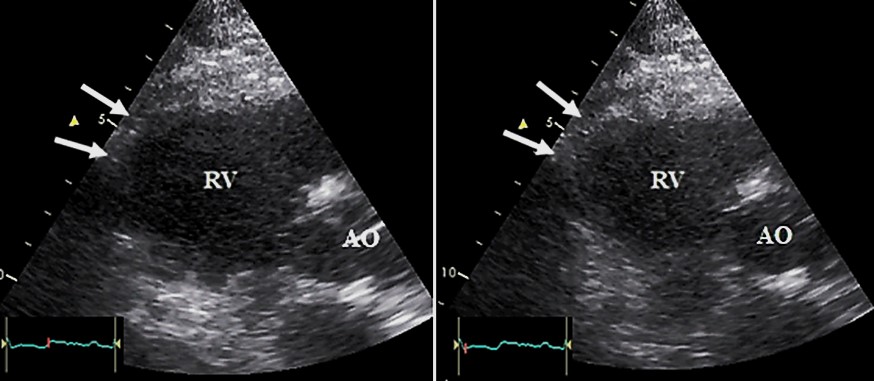

Supplement: Supplementary file 1 [file diagnostics-13-02194-s001.zip › figure S5-case 7.jpg]

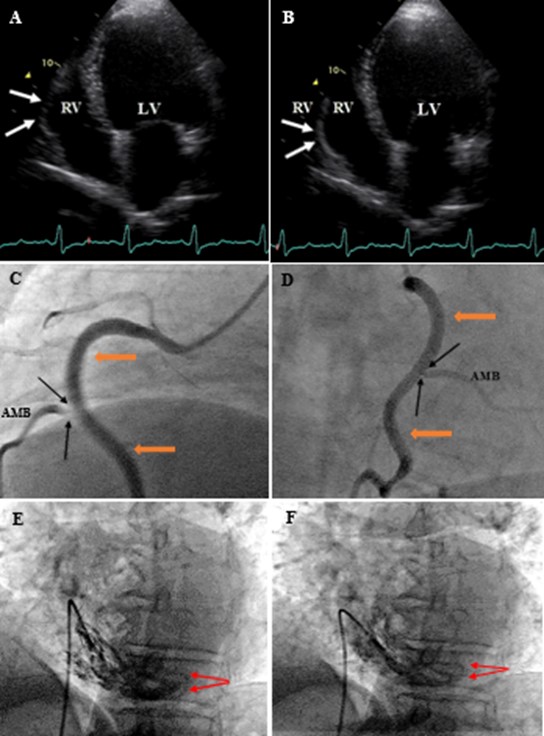

Supplement: Supplementary file 1 [file diagnostics-13-02194-s001.zip › figure S6-case 8.jpg]

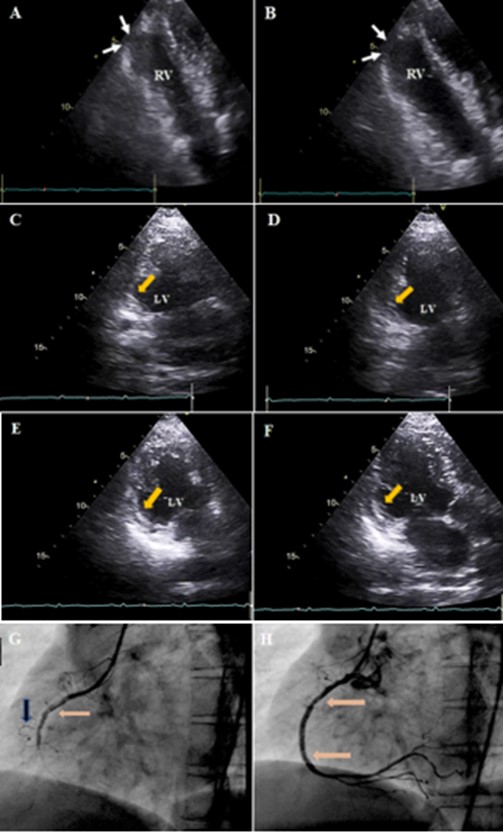

Supplement: Supplementary file 1 [file diagnostics-13-02194-s001.zip › figure S7-case 9.jpg]

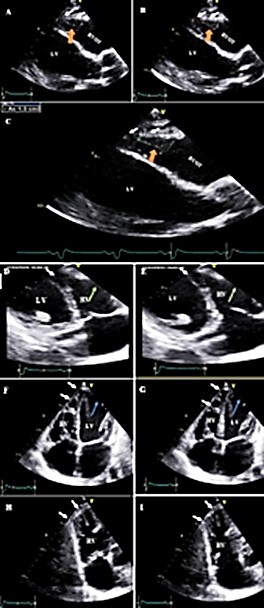

Supplement: Supplementary file 1 [file diagnostics-13-02194-s001.zip › figure S8-case 10.jpg]

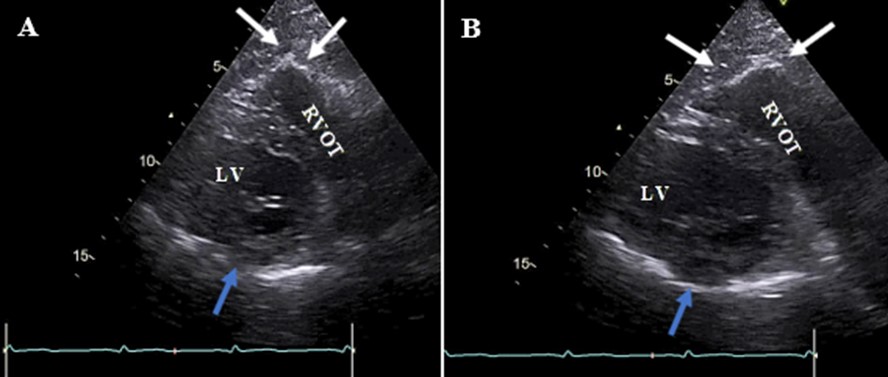

Supplement: Supplementary file 1 [file diagnostics-13-02194-s001.zip › figure S9-case 11.jpg]
